# Thermostabilization of the Human Serotonin Transporter in an Antidepressant-Bound Conformation

**DOI:** 10.1371/journal.pone.0145688

**Published:** 2015-12-22

**Authors:** Evan M. Green, Jonathan A. Coleman, Eric Gouaux

**Affiliations:** 1 Vollum Institute, Oregon Health & Science University, Portland, Oregon, United States of America; 2 Howard Hughes Medical Institute, Oregon Health & Science University, Portland, Oregon, United States of America; University of Bern, SWITZERLAND

## Abstract

Serotonin is a ubiquitous chemical transmitter with particularly important roles in the gastrointestinal, cardiovascular and central nervous systems. Modulation of serotonergic signaling occurs, in part, by uptake of the transmitter by the serotonin transporter (SERT). In the brain, SERT is the target for numerous antidepressants including tricyclic antidepressants and selective serotonin reuptake inhibitors (SSRIs). Despite the importance of SERT in human physiology, biochemical, biophysical and high-resolution structural studies have been hampered due to the instability of SERT in detergent micelles. To identify a human SERT (hSERT) construct suitable for detailed biochemical and structural studies, we developed an efficient thermostability screening protocol and rapidly screened 219 mutations for thermostabilization of hSERT in complex with the SSRI paroxetine. We discovered three mutations—Y110A, I291A and T439S –that, when combined into a single construct, deemed TS3, yielded a hSERT variant with an apparent melting temperature (*Tm*) 19°C greater than that of the wild-type transporter, albeit with a loss of transport activity. Further investigation yielded a double mutant—I291A and T439S—defined as TS2, with a 12°C increase in *Tm* and retention of robust transport activity. Both TS2 and TS3 were more stable in short-chain detergents in comparison to the wild-type transporter. This thermostability screening protocol, as well as the specific hSERT variants, will prove useful in studies of other integral membrane receptors and transporters and in the investigation of structure and function relationships in hSERT.

## Introduction

The human serotonin transporter (hSERT) belongs to the family of neurotransmitter sodium symporters (NSSs) that terminate neurotransmission by clearing neurotransmitters from the synaptic cleft [1]. hSERT pumps serotonin from the synaptic cleft and into presynaptic neurons by the thermodynamically favorable symport of Na^+^ and Cl^-^ ions. Serotonin is a neurotransmitter that is important in a number of neurological signaling pathways. Dysregulation of serotonergic signaling has been implicated in disorders including depression and anxiety [2]. Accordingly, hSERT is a primary target for antidepressant drugs such as the selective serotonin reuptake inhibitors (SSRIs), including paroxetine, citalopram, sertraline and fluoxetine. Structural information about the NSS family was first revealed by the x-ray crystal structure of the *Aquifex aeolicus* leucine transporter (LeuT) [3]. Subsequent structures of LeuT in multiple conformations have contributed greatly to the understanding of the architecture and transport cycle of the NSS family [4–8]. NSS proteins are characterized by 12 transmembrane (TM) helices, a large glycosylated extracellular loop between TM3 and TM4 in the mammalian orthologs [2] and centrally located substrate and ion binding sites located approximately midway across the membrane bilayer [3,9]. Members of the NSS family, which includes the serotonin, dopamine and norephinephrine monoamine transporters, are the targets for therapeutics of numerous disorders as well as drugs of addiction [10]. Recently, structures of the *Drosophila melanogaster* dopamine transporter (dDAT) have revealed novel structural elements not seen in LeuT, including a bound cholesterol molecule that may have a role in allosteric control of the transporter [9,11,12]. To allow us to probe relationships between structure, function and inhibition in hSERT, we set out to generate a hSERT construct suitable for structural, biochemical and biophysical studies.

Though structure determination of eukaryotic membrane proteins continues to lag significantly behind other targets, the use of the lipidic cubic phase, new detergents, antibody fragments, T4 lysozyme fusions and thermostabilized constructs have increased the number of structures determined by X-ray crystallography [13–16]. Even with these technological breakthroughs, however, eukaryotic membrane protein crystallization and structure determination remains difficult as highlighted by the fact that <1% of the structures deposited the Protein Data Bank are from these elusive targets [17]. In many cases, including a number of G-protein coupled receptors and dDAT, the use of conformationally-specific thermostabilizing mutations were necessary for crystallization [11,18]. Thermostabilizing mutations are beneficial for multiple reasons: they can increase the yield of well-behaved monodisperse protein, increase stability in short chain detergents and can stabilize conformational states that are difficult to crystallize [19].

Previous work identified a number of mutations that increase the thermostability of *Rattus norvegicus* SERT (rSERT) bound to the high affinity radiolabeled cocaine analog [^125^I]RTI-55 using alanine-scanning mutagenesis and gel-filtration-based radioligand binding assays [20]. Although rSERT and hSERT have 92% sequence identity, they exhibit different inhibitor binding affinities [21]. In particular, rSERT has a 4–5 fold lower affinity for tricyclic antidepressants including imipramine, clomipramine and desipramine. We decided on paroxetine as a candidate ligand to measure thermostabilization and cocrystallization due to its high affinity and clinical relevance. The gel-filtration-based thermostability screen described by Abdul-Hussein *et al* was not suitable to study paroxetine binding due to high background arising from non-specific binding of paroxetine to detergent micelles. To facilitate the structure determination of hSERT, we developed a technique to screen for thermostabilizing mutations in a ligand-bound conformation. Here we describe this scintillation proximity assay (SPA) based technique used to identify conformation-stabilizing mutations in a high-throughput fashion as well as a highly thermostabilized hSERT construct suitable for structural work [22,23].

## Materials and Methods

### Reagents

Detergents and cholesteryl hemisuccinate (CHS) were purchased from Anatrace. Unlabeled and radiolabeled ligands were purchased from Sigma-Aldrich and PerkinElmer, respectively.

### Cloning

Human serotonin transporter cDNA was generously provided by Randy Blakely [24] and cloned into the BacMam expression vector as a fusion protein with a C-terminal Thrombin-GFP-StrepII-H_10_ tag. hSERT was further modified by a truncation of 72 and 12 amino acids from the N- and C-terminus, respectively.

Single point mutations were generated by site-directed mutagenesis. Codons for mutated residues were optimized for expression in HEK293S cells. In total 219 point mutants were made in the minimal construct background, 180 of which were made by the QB3 MacroLab at the University of California, Berkeley.

### Cell culture and transient transfection

Adherent HEK293S GnTl^-^ cells grown in Dulbecco’s Modified Eagle Medium with 10% fetal bovine serum at 37°C were transiently transfected using PolyJet according to the manufactures protocol (0.1 μg or 1 μg/well for 96-well and 6-well format, respectively) at 90% confluency. Sixteen hours post transfection the media was replaced and supplemented with 10 mM sodium butyrate, a histone deacetylase inhibitor that enhances recombinant protein expression [25,26]. Two days post transfection the media was aspirated and the cells were used immediately or stored at -80°C.

### High-throughput thermostability screen

Each hSERT mutant was expressed in four wells with approximately 50,000 HEK293S cells/well. After expression, cells were incubated with TBS (20 mM Tris pH 8.0, 150 mM NaCl) supplemented with 80 nM unlabeled paroxetine in 25 μL for 5 minutes at room temperature. Cells were solubilized for one hour at room temperature without shaking with 4 mM C12M (n-dodecyl β-D-maltoside), 0.5 mM CHS, 1 mM phenylmethanesulfonyl fluoride (PMSF), 0.05 mg/mL aprotinin, 2 μg/mL pepstatin A and 2 μg/mL leupeptin in 50 μL. Following solubilization [^3^H]paroxetine was added and the volume was brought to 100 uL with a final concentration of 20 nM [^1^H]paroxetine, 20 nM [^3^H]paroxetine (15.5 Ci/mmol specific activity, working concentration), 2 mM C12M, 0.25 mM CHS, 0.05% bovine serum albumin and 1 mg/mL Cu-YSi SPA beads (PerkinElmer). Radioligand binding was measured using a MicroBeta Trilux Scintillation Detector (PerkinElmer) at room temperature with a one-minute count time per well. Plates were counted until the total counts plateaued (approximately 36 hours), after which plates were heated for 15 minutes in an Eppendorf ThermoMixer C with a heated lid and counted again. Nonspecific binding was determined by the addition of 100 μM sertraline to a single well for each mutant. Specific counts were determined by averaging three wells transfected with a single mutant and subtracting the average nonspecific binding of all mutants on a single plate. The heating step was repeated until all mutants had low specific counts. Apparent melting temperatures (*T*_*m*_) were determined by a non-linear fit to a Boltzmann sigmoidal function with Prism 6 (GraphPad).

### Traditional thermostability assay

Transfected HEK293S cells grown in 6-well plates were harvested (two wells/mutant, approximately 2 million cells total) and incubated with 80 nM unlabeled paroxetine for 5 minutes at room temperature in 1 mL TBS. Cells were solubilized with 4 mM C12M, 0.5 mM CHS, 1 mM PMSF, 0.05 mg/mL aprotinin, 2 μg/mL pepstatin A and 2 μg/mL leupeptin in 2 mL at 4°C with rotation. Cell debris was removed by centrifugation (18,000 x g for two minutes). The supernatant was split into eight fractions and heated for 30 minutes using a thermocycler. After heating, each sample was aliquoted into four wells in a 96-well plate with 50 μL per well. [^3^H]Paroxetine was added and the volume was brought to 100 uL with a final concentration of 20 nM [^1^H]paroxetine, 20 nM [^3^H]paroxetine (15.5 Ci/mmol specific activity, working concentration), 2 mM C12M, 0.25 mM CHS, 0.05% bovine serum albumin and 1 mg/mL Cu-YSi SPA beads. Radioligand binding was measured using a MicroBeta Trilux Scintillation Detector at room temperature with a one-minute count time per well. Plates were counted until the total counts plateaued. Nonspecific binding was determined by the addition of 100 μM sertraline to a single well for each mutant. Specific counts were determined by averaging three wells for each sample and subtracting the average nonspecific binding of all mutants on a single plate. Apparent melting temperatures (*T*_*m*_) were determined by a non-linear fit to a Boltzmann sigmoidal function with Prism 6.

### [^14^C]serotonin uptake assay

HEK293S cells were transduced with baculovirus in 50 ml suspension cultures. One day post-infection, 175,000 cells were added to 96-well cytostar T plates (Perkin Elmer) and diluted to 200 μl in DMEM supplemented with 10% FBS. Cells were allowed to attach for 3 hrs and then washed with 25 mM HEPES-Tris pH 7.0, 130 mM NaCl, 5.4 mM KCl, 1.2 mM CaCl2, 1.2 mM MgSO4, 1 mM ascorbic acid, and 5 mM glucose. For control samples, 10 μM paroxetine was added and allowed to bind to hSERT for 5 min at room temperature. Uptake of 10 μM [^14^C]serotonin (9.6 mCi/mmol specific activity, working concentration) was followed in the MicroBeta Trilux at room temperature. Uptake after 35 minutes was fit to a Michaelis-Menten equation in Prism 6 to determine *K*_M_ values.

### Ligand binding assay

Ligand binding experiments were carried out by adding HEK293S membranes containing SERT to a final concentration of 2 nM in 1 ml of TBS with 0.01–10 nM [^3^H]paroxetine (15.5 Ci/mmol specific activity, working concentration). Reactions were rotated at room temperature for several hours followed by filtering through a glass microfiber filter prewet with 0.4% polyethylenimine in TBS. Membranes were washed 3x with 4 ml of cold TBS followed by liquid scintillation counting. Data was fit to a single-site binding curve accounting for ligand depletion and non-specific binding in Prism 6 [27].

## Results

### Mutant selection

**Fig 1:**
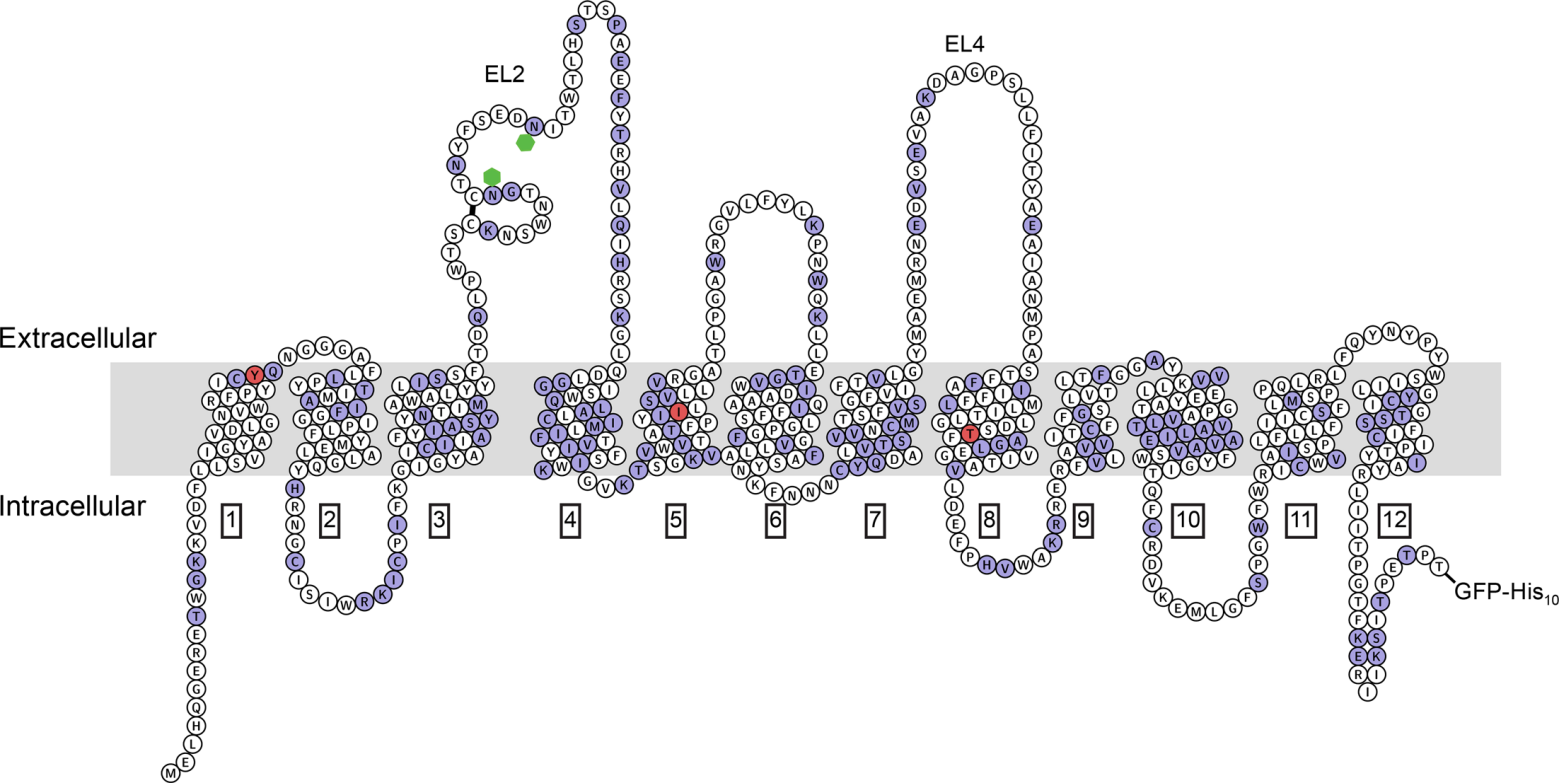
“Snake” plot showing mutated residues. Residues in purple were mutated in the thermostability screen. Of the tested constructs three were combined to generate a thermostabilized hSERT construct Y110A, I291A and T439S (red). Transmembrane helices are labeled in black boxes and extracellular loops are labeled as EL2 and EL4. hSERT also has a disulfide bond and two predicted glycosylated asparagine residues, which are demarcated by a black line and green hexagons, respectively. Topology and position of the transmembrane helices were determined by sequence alignment to the structure of dDAT.

To identify positions that could thermostabilize hSERT, we performed sequence alignment of hSERT with dDAT (PDB ID: 4M48, 49% sequence identity) and manually selected 219 point mutations at 160 different residues based on the following criteria: 1) large charged residues in highly charged clusters, especially those on the surface; 2) kinked regions adjacent to α-helices involved in conformational changes; 3) branched side chains in α-helices; 4) residues lining solvent-exposed cavities; 5) post-translationally modified residues; 6) residues that are different in LeuT; 7) mutations known to thermostabilize rSERT and dDAT. The selected mutations are distributed across all 12 transmembrane segments (Fig 1).

### High-throughput SPA-based thermostability screen

**Fig 2:**
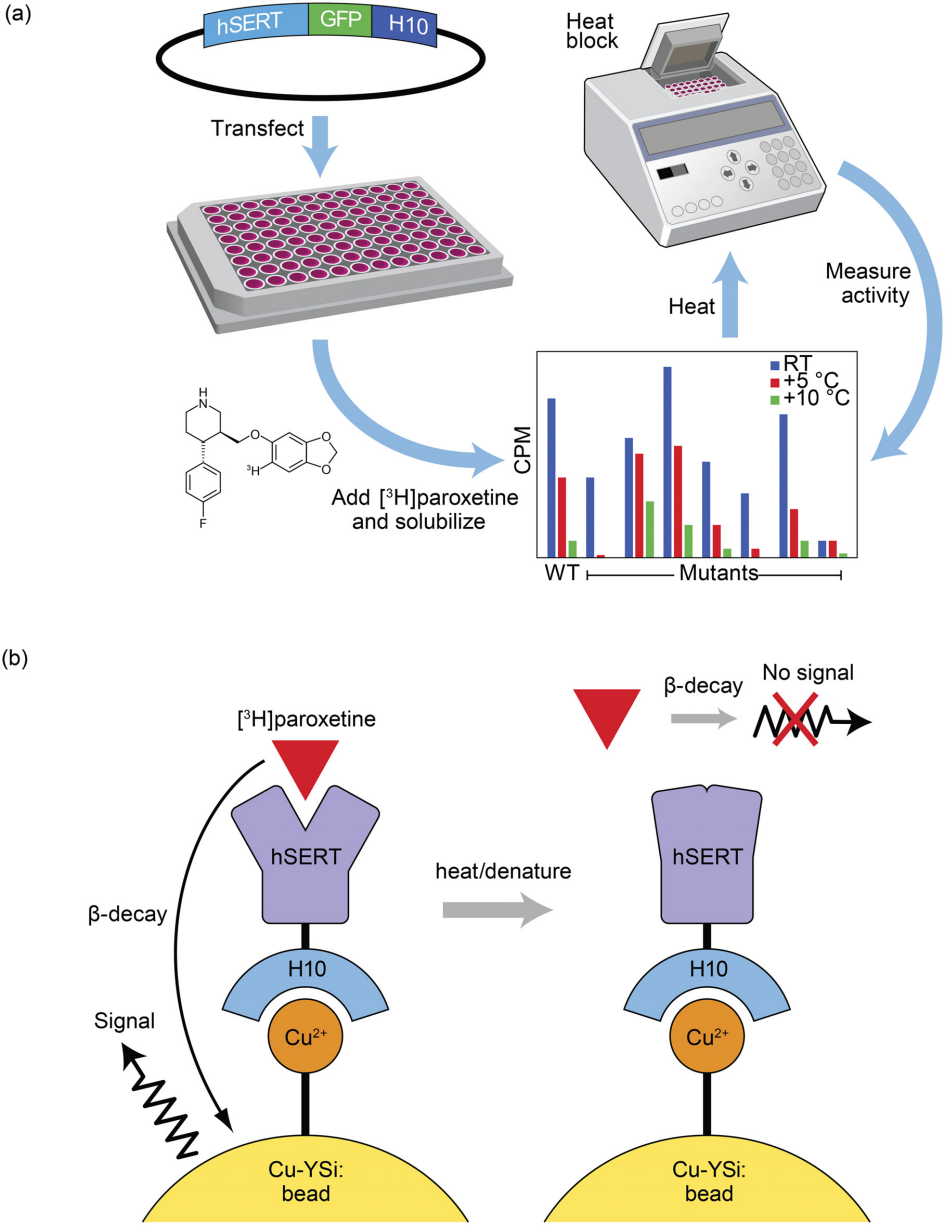
Experimental design. **a**, HEK293S cells were transiently transfected with the plasmid containing the encoded hSERT-GFP-H_10_ fusion protein. After expression for two days, paroxetine was added and the cells were solubilized. The amount of bound [^3^H]paroxetine is measured using Cu-YSi SPA beads. The sample was repetitively heated with the amount of bound paroxetine measured at each step. Constructs with high relative amounts of bound paroxetine after heating represent thermostabilizing mutations. **b**, Scheme of SPA step. Prior to heating, hSERT was bound to the Cu-YSi beads via the H_10_ tag. Any bound [^3^H]paroxetine which is in close proximity to the bead which undergoes β-decay can cause a detectable scintillation event. After heating, the protein is denatured and unable to bind [^3^H]paroxetine. Unbound [^3^H]paroxetine is not close enough to the Cu-YSi bead to cause a scintillation event.

Crystallization of membrane proteins is a challenging task that often requires multiple rounds of construct optimization. Generating and testing a large library of single-point mutations to enhance stability is a time consuming task that is often a bottleneck in the crystallization process. To facilitate the rapid screening of hSERT mutants we set out to develop a high-throughput SPA-based thermostability screen that can rapidly screen for constructs with enhanced thermostability in a ligand-bound conformation (Fig 2).

We generated a C-terminally tagged hSERT-GFP-His_10_ construct to allow us to monitor hSERT expression via epifluorescence and fluorescence-detection, size-exclusion chromatography (FSEC) [28]. The N- and C-termini were truncated by 72 and 12 residues, respectively, based on deletions employed in the recently crystallized dDAT [9,11,12]. The resulting ‘minimal’ hSERT construct exhibited a relatively sharp peak for the SERT-GFP fusion (S1 Fig) [28], indicative of a monodisperse membrane protein.

With a well-behaved minimal construct in hand, we next focused on generating a hSERT-SSRI complex with enhanced thermostability. A previous thermostabilization screen of rSERT used a single-point gel-filtration-based thermostability screen to identify mutations that increased the thermostability of [^125^I]RTI-55 bound transporter [20]. Unfortunately, this method was not suitable for screening of the hSERT-paroxetine complex due to high non-specific binding to detergent micelles. We therefore set out to develop a new method to screen for thermostabilized SSRI-bound hSERT. A SPA-based method was chosen because the assay allows for multiple measurements, requires minimal sample manipulation and is compatible with several radioisotopes.

hSERT was expressed in HEK293S GnTl^-^ cells by transient transfection in 96-well plates (Fig 2) [25]. Cells were incubated with paroxetine prior to solubilization with detergent to increase the stability of the transporter. If cold paroxetine was not added prior to solubilization, hSERT was incapable of subsequently binding [^3^H]paroxetine. After solubilization, Cu-YSi SPA beads and [^3^H]paroxetine were added. After equilibration, hSERT was capable of binding paroxetine at room temperature for up to a week as assessed by monitoring counts over time. The long-term stability of paroxetine binding at room temperature and the homogeneous nature of the SPA method made it possible to heat samples after equilibration at room temperature. After equilibration the 96-well plates were heated for 15 minutes at successively higher temperatures ranging from 43–55°C on a 96-well plate heating block. The amount of bound [^3^H]paroxetine was determined by specific CPM after each successive heating step; hSERT which becomes denatured due to the heating step can no longer bind [^3^H]paroxetine as indicated by the reduced specific counts. Samples were heated repetitively until there was minimal specific paroxetine binding.

**Fig 3:**
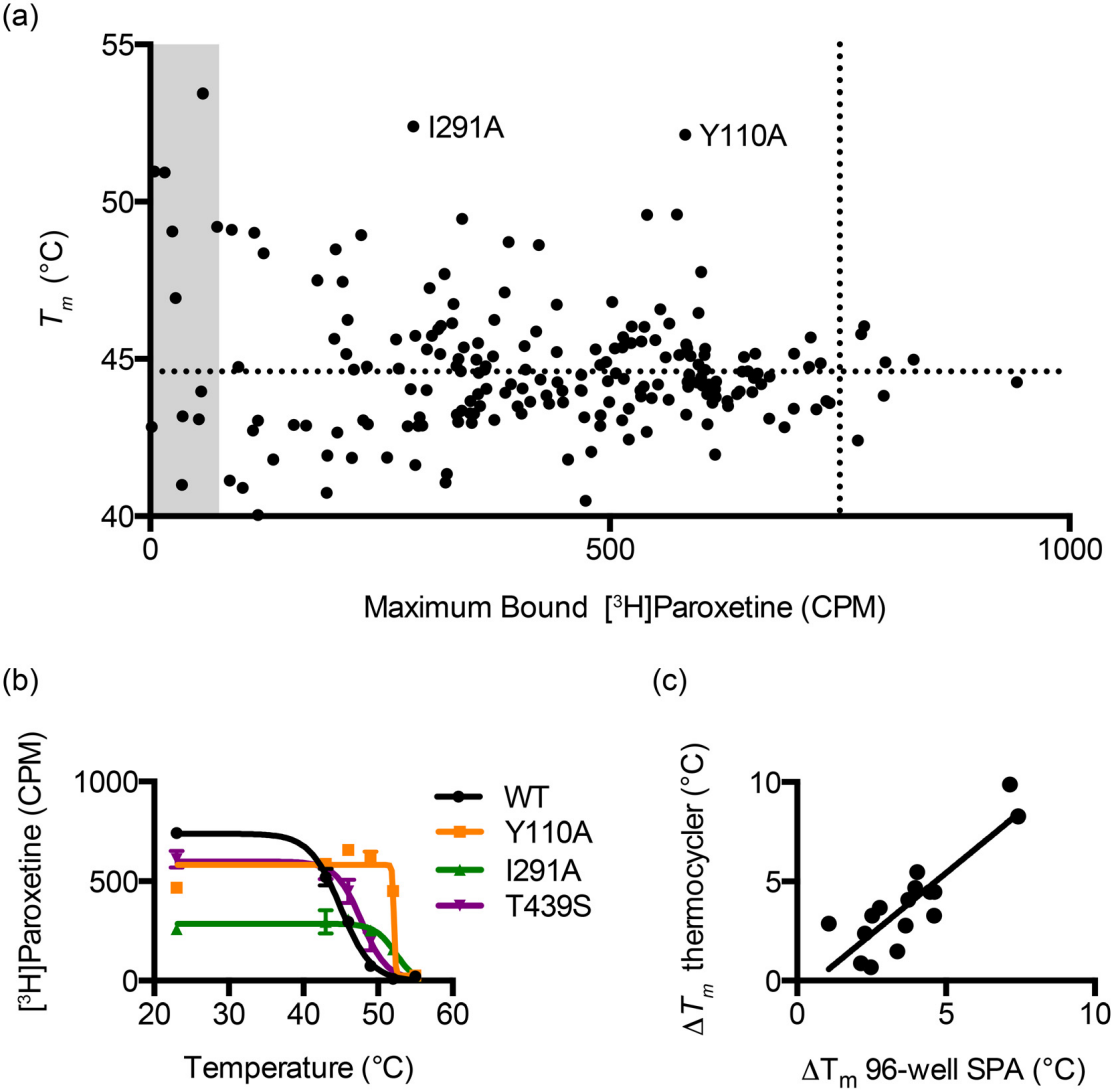
Results from paroxetine-bound thermostability screening assay. **a**, Comparison of maximum bound [^3^H]paroxetine and measured melting temperature (*Tm*). Dotted lines represent values for wild-type hSERT. The gray region represents mutants that have less than 10% of paroxetine binding compared to wild-type hSERT and also have large errors in *Tm* measurements. **b**, Curve fits for wild-type hSERT and the two most stabilizing mutants, Y110A and I291A. **c**, Comparison of *Tm* values from 96-well format single-point assay and traditional melting curve measurement. All CPM values are specific CPM. Error bars represent standard errors of the mean.

The SPA-based screening method was used to measure the relative thermostability of 219 hSERT single-point mutants in the presence of paroxetine (Fig 3). The repetitive heating steps made it possible to determine the apparent melting temperature (*Tm*) for all of the mutants tested, except for approximately 10% of mutants with little or no paroxetine binding at room temperature (<75 specific CPM). The top mutants from the high-throughput screen were expressed in 6-well plates and their *Tm* values were redetermined using a traditional thermostability assay with samples heated in a thermocycler prior to the addition of [^3^H]paroxetine and the *Tm* values were in good agreement between the two methods (Fig 3c). The maximum difference between the two methods was 2.5°C. Of the 16 single mutants that were retested, none had a *Tm* lower than that of the wild-type transporter, suggesting a low frequency of false positives.

The new single-point repetitive heating SPA method has several advantages over previous methods: it reduces the likelihood of finding false positives by determining a complete melting curve, it uses little radioactive material and requires minimal sample manipulation due to the use of a single 96-well plate. Importantly, the new method is compatible with hydrophobic hSERT inhibitors, such as paroxetine, that were not amenable to gel-filtration-based thermostability screening. A single individual screened all 219 mutants in two weeks.

### Testing mutants

**Fig 4:**
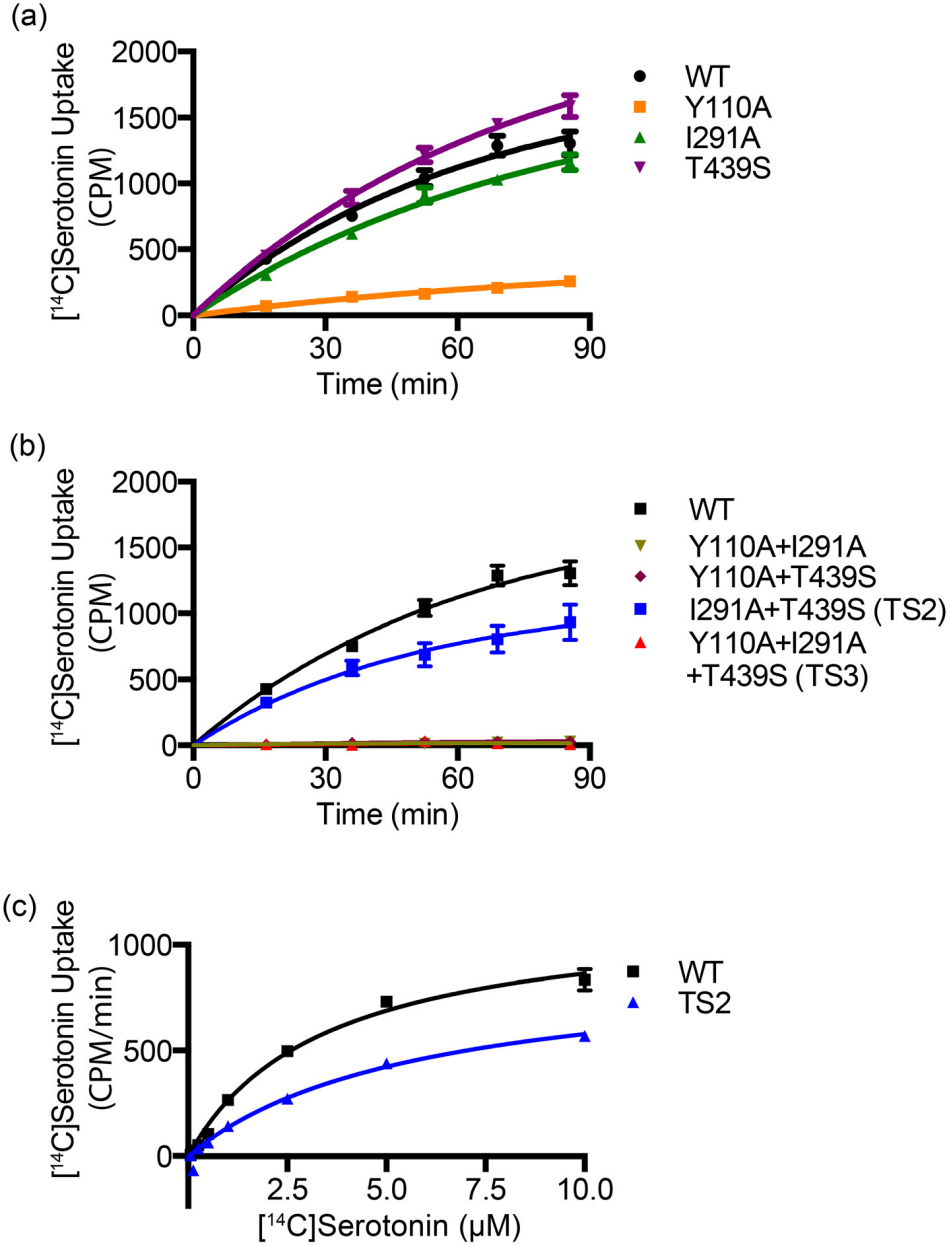
[^14^C]Serotonin uptake measurements. **a**, The [^14^C]serotonin uptake of single mutants and **b**, combined mutants. **c**, Michaelis-Menten plot for [^14^C]serotonin uptake of wild-type and TS2 mutant gave a *K*_M_ of 3.4 and 5.8 μM, respectively. Error bars represent standard errors of the mean.

Three mutants were selected for additional screening based on their increased thermostability. These mutants and their apparent *Tm*s are Y110A (52°C), I291A (50°C) and T439S (46°C). Of the three single mutants that were tested, I291A and T439S had serotonin uptake similar to wild-type hSERT while Y110A had significantly reduced serotonin uptake (Fig 4). Combinations of any of the mutations with Y110A eliminated serotonin uptake. Serotonin uptake by the wild-type transporter was shown to be saturable with a *K*_M_ of 3.4 μM (2.1–4.7 95% confidence interval, CI). The I291A/T439S double mutant had a slightly higher *K*_M_ of 5.8 μM (1.2–10.4 95% CI). Empty plasmid controls did not have any measurable [^14^C]serotonin uptake.

**Fig 5:**
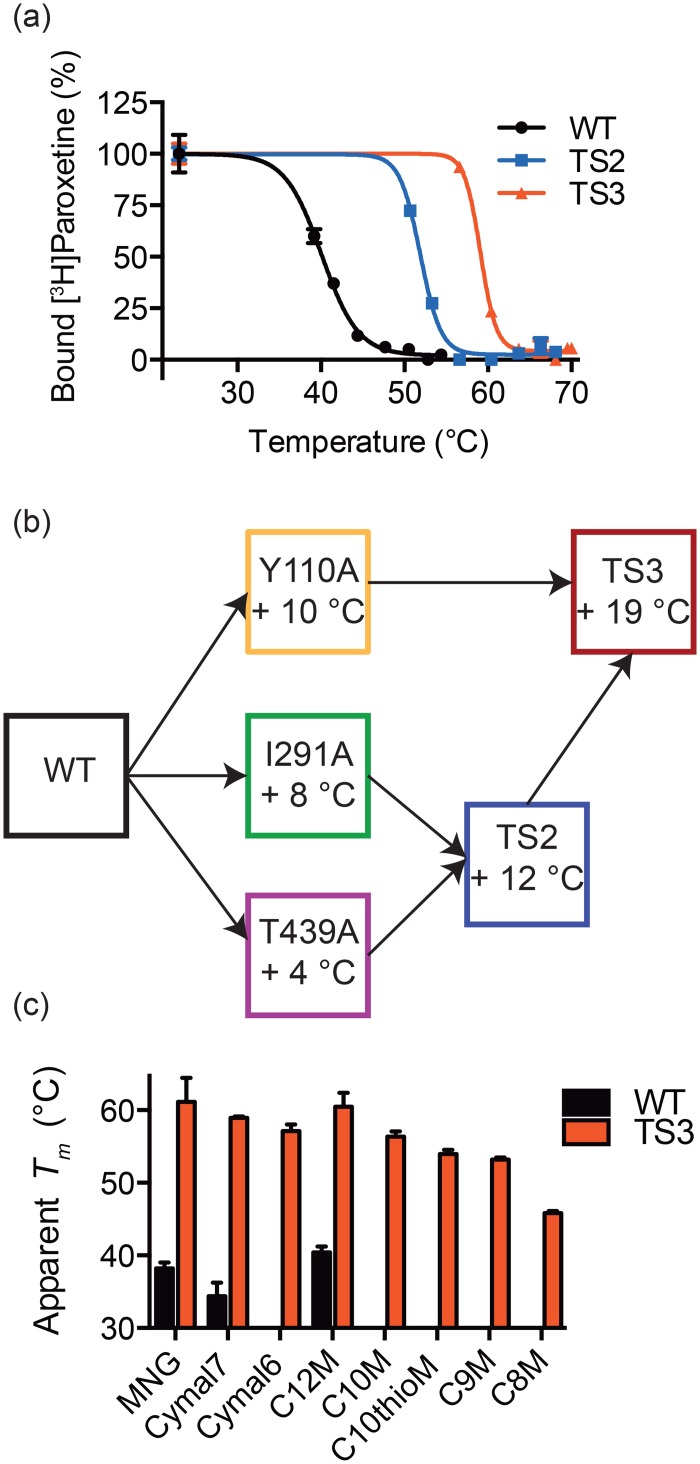
Increased thermostability and detergent stability. **a**, The TS2 and TS3 mutations increase the stability of hSERT by 12 and 19°C, respectively. **b**, Strategy for combining mutants. TS2 and TS3 were made to increase hSERT stability. **c**, TS3 mutations increase the stability of hSERT in a wide range of detergents (MNG: lauryl maltose neopentyl glycol, C12M: n-dodecyl-β-D-maltopyranoside, DM: n-decyl-β-D-maltopyranoside, C10thioM: n-decyl-β-D-thiomaltopyranoside, C9M: n-nonyl-β-D-maltopyranoside, C8M: n-octyl-β-D-maltopyranoside) Columns without bars indicate it was not possible to measure the thermostability, potentially due to stability below room temperature. Error bars represent standard errors of the mean.

The mutants were combined to make the functional double mutant, TS2 (I291A/T439S), and a non-functional triple mutant, TS3 (Y110A/I291A/T439S) (Fig 5). TS2 showed an additive increase in thermostability with a 12°C increase in *Tm* over the wild-type transporter. The absolute difference in the *Tm* values measured by the traditional thermostability assay compared to the repetitive heating experiments are likely due to a higher concentration of C12M at the time of heating (4 mM vs. 2 mM) and increased heating time (30 vs. 15 minutes). The addition of the Y110A mutation to TS2 further increased the thermostability to 59°C, a 19°C increase over the wild-type transporter. TS3 was more stable than the wild-type transporter in a wide variety of detergents, including short chain detergents such as C10M and C8M, detergents that often facilitate membrane protein crystallization (Fig 5).

**Fig 6:**
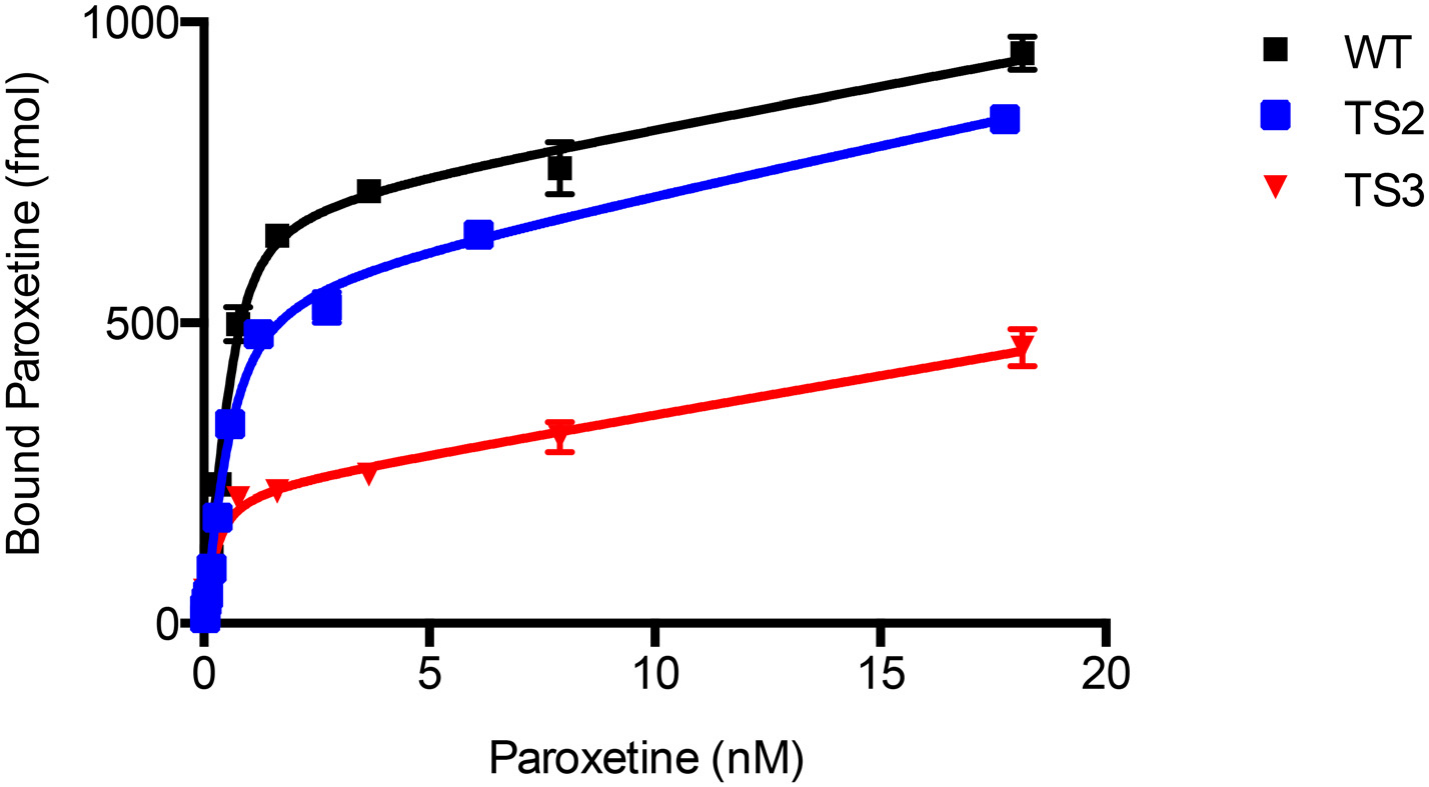
Paroxetine binding affinity of TS3 and wild-type hSERT. The paroxetine binding affinity of wild-type hSERT and TS3 was measured by membrane filter binding. Total counts were fit to a one site model accounting for ligand depletion due to the high affinity of the ligand, and representative curves are shown. The paroxetine *K*_*d*_ was 0.14, 0.22 and 0.103 for wild-type hSERT, TS2 and TS3, respectively. Error bars represent standard errors of the mean.

To understand if increased stability correlated with increased paroxetine affinity we performed filter-binding experiments (Fig 6). The wild-type transporter had a binding affinity for paroxetine of 0.14 nM (0.11–0.16 95% CI), similar to the reported value for platelet membranes of 0.08 nM [29]. However, TS2 had a slightly lower affinity of 0.22 nM (0.16–0.28 95% CI) while TS3 had a similar affinity of 0.10 nM (0.05–0.16 95% CI). The non-flat plateau is due to non-specific binding of paroxetine to the membranes, which was not diminished even with three washes. The data suggests that there is not a significant correlation between thermostability and increased paroxetine affinity. The lack of a significant increase in the affinity of the thermostabilized constructs to paroxetine is similar to the thermostabilized rSERT which showed similar affinity to RTI-55 compared to the wild-type transporter [20].

## Discussion

Thermostabilization strategies have aided structure determination of many important eukaryotic membrane transporters and receptors. However, preexisting screening approaches were not suited to hSERT due the extremely labile behavior of the unliganded transporter following extraction from the membrane bilayer. We thus developed a high-throughput SPA-based thermostability assay designed to stabilize hSERT upon membrane extraction, an assay that should be widely useful for other unstable membrane proteins. Using our assay we screened 219 single point mutants and discovered that, by combining single thermostabilizing mutants to form double (TS2) or triple mutants (TS3), we were able to increase the *Tm* of hSERT by 12 and 19°C, respectively.

Previous strategies used for the screening of mutations and conditions that increase thermostability have used FSEC, thiol-reactive dyes and gel-filtration-based radiolabeled ligand binding [15,30,31]. The new SPA-based thermostability assay is high-throughput, highly specific and can screen for thermostabilization in a ligand specific manner. The protocol is complementary to the current methods and has several advantages over previous screening assays. Specifically, it is possible to screen with high affinity hSERT inhibitors that were not possible due to high background in the gel-filtration radiolabeled ligand binding methods. The method also allows direct determination of a construct’s melting curve, yielding more information than a single heating point and potentially reducing the rate of false positives. Finally, our screening strategy requires minimal sample manipulation, which reduces radioactive waste and makes it amenable for automated high-throughput screening.

**Fig 7:**
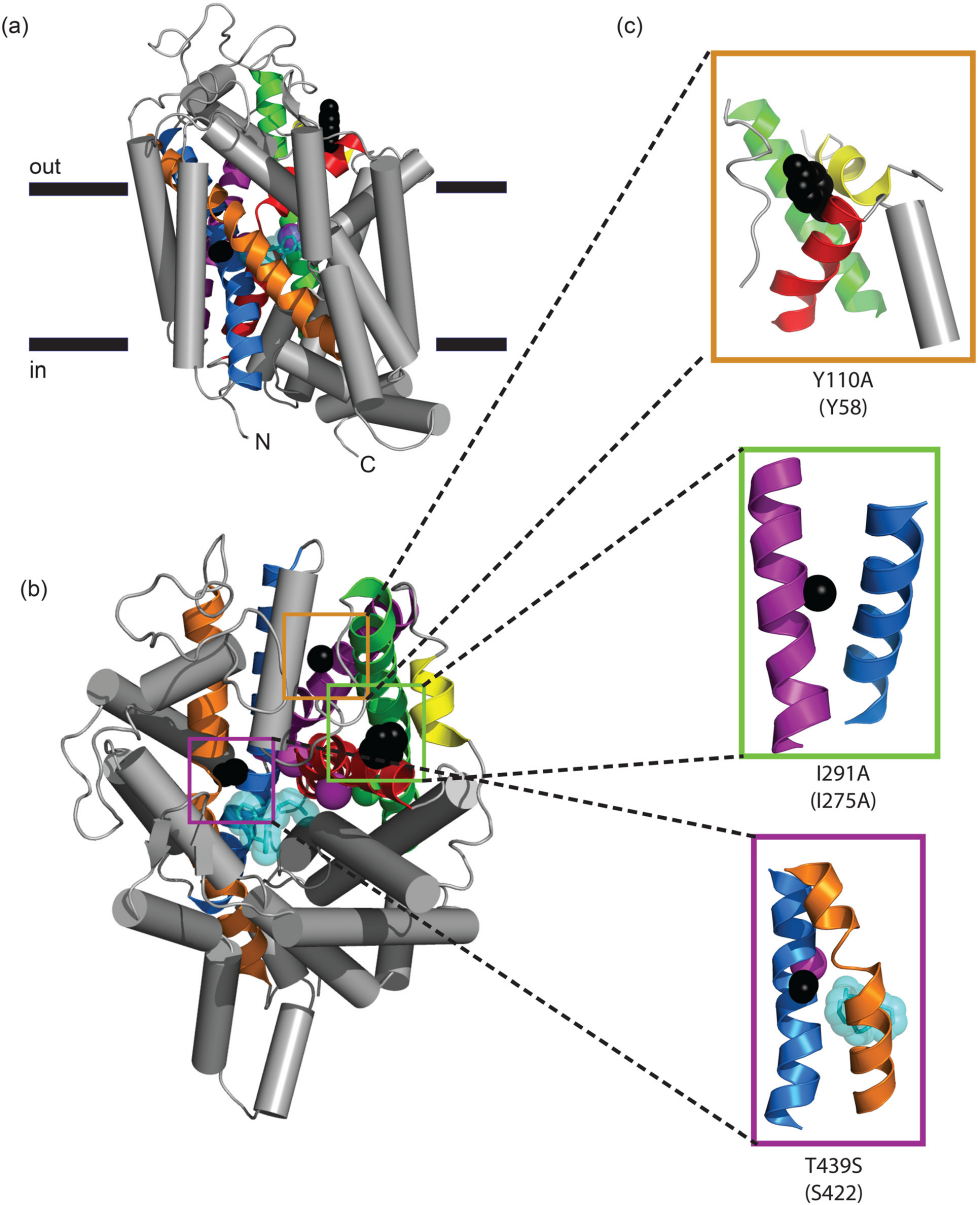
The position of hSERT mutations in the dDAT structure. The position of the three characterized thermostabilizing mutations are shown superimposed on the homologous residues from the dopamine transporter structure (PDB ID: 4M48). The overall positions are shown from side (**a**) and top (**b**) views. **c**, Close up of view of Y110A (top), I291A (middle), and T439S (bottom) are shown on residues Y58, A275, and S422 from dDAT, respectively. Side chains are shown as black spheres, sodium atoms are magenta spheres, chloride atoms are green spheres, nortriptyline is cyan.

Using the optimized thermostability screen described above, three mutations were identified and combined to generate the highly thermostabilized construct, TS3. The three mutations are predicted to be located in distinct regions of hSERT based on sequence alignment to dDAT (Fig 7). The most stabilizing mutation, Y110A, raises the *Tm* by 10°C and is located at the top of TM1b. The corresponding residue in dDAT is Y58. Structural studies and DEER measurements of LeuT show that TM1 undergoes significant structural rearrangements during the substrate transport cycle [4,5,32]. It is tempting to speculate that perturbations within this critical location shift the conformational equilibrium towards an outward-facing state that abolishes serotonin uptake. The next most thermostabilizing mutation, I291A, increases the *Tm* by 8°C and is in TM5, facing TM8. This residue is homologous to residue 275 in dDAT, previously identified as a site of thermostabilization in dDAT [11], and is in close proximity to a cholesterol molecule that might have a functional role in the transport cycle. The final mutation, T439S, is found in TM8 near both Na2 and the ligand-binding site in dDAT. Mutation to serine instead of alanine was chosen at this site since the corresponding residue in both dDAT and LeuT is serine. Finally, we note that the mutations that confer stability in the paroxetine-bound form do not correspond to the mutations that stabilized rSERT in a cocaine-bound conformation. Subtle differences in the sequence and conformation of hSERT seem to require entirely different mutations to achieve thermostabilization. This highlights the need for conformationally specific, high-throughput screening techniques like the assay presented here.

Using the thermostability screen described above we were able to rapidly screen over 200 mutants. The initial positive hits were further characterized and used to generate two hSERT constructs that are highly stable and suitable for crystallization screening and other biochemical or biophysical studies. Crystallization of hSERT with paroxetine will increase our understanding of both SSRI activation and binding as well as provide useful information into how the various monoamine transporters are able to achieve substrate specificity. We believe that the new SPA-based thermostabilization method described above, given its simplicity, is broadly applicable to screen mutations that stabilize specific conformations in many membrane protein targets.

## Supporting Information

S1 Fig Fluorescence size-exclusion chromatography of hSERT mutants. Traces from single (**a**) and multiple (**b**) hSERT mutants. Peaks are labeled as * void; # SERT-GFP; ^ cleaved GFP.
(TIF)
