# Supplementary figures and images for: Thermostabilization of the Human Serotonin Transporter in an Antidepressant-Bound Conformation
Source: PLoS One. 2015 Dec 22;10(12):e0145688. doi: 10.1371/journal.pone.0145688 (PMC4687910; doi:10.1371/journal.pone.0145688)

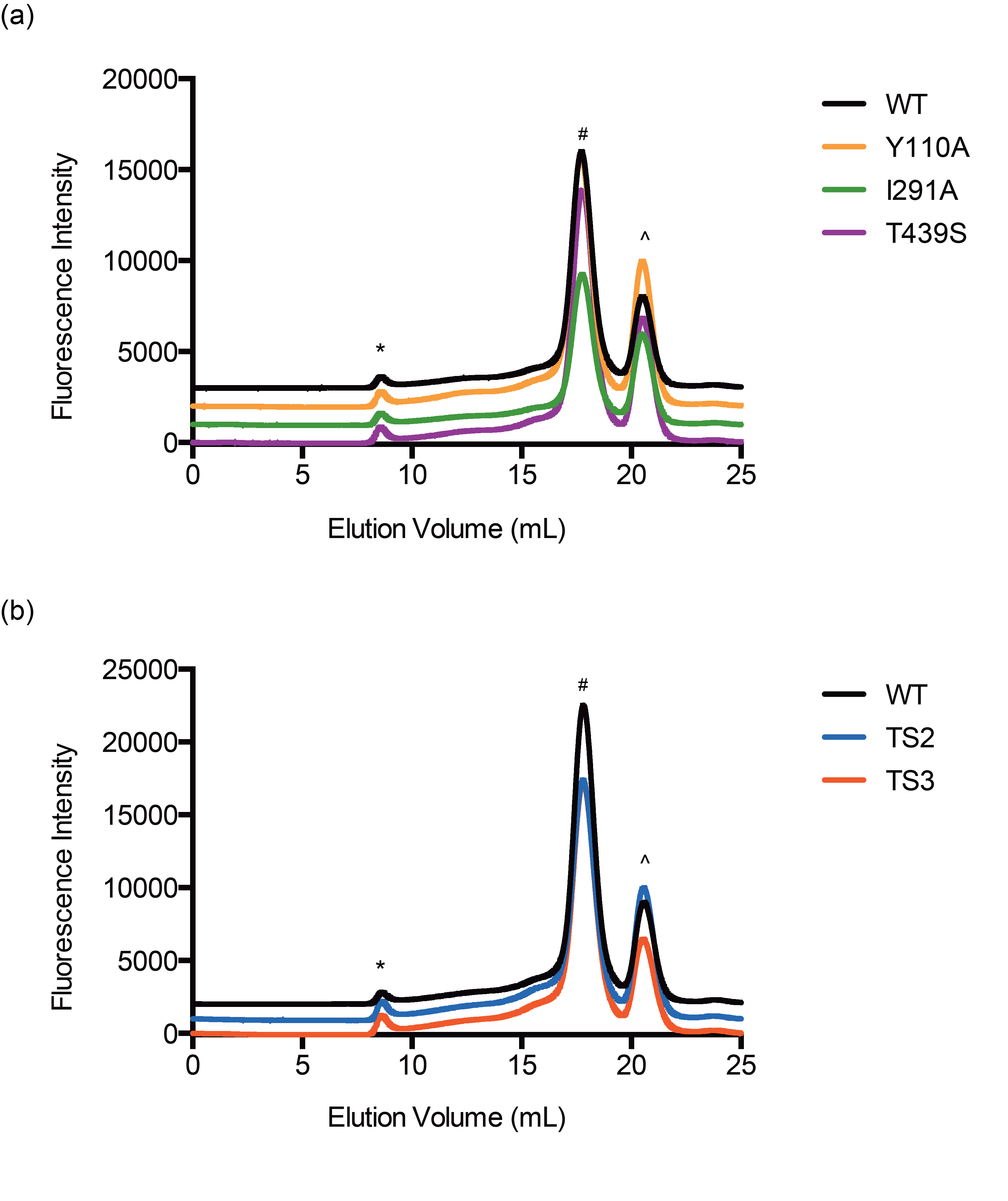

Supplement: S1 Fig — Traces from single (a) and multiple (b) hSERT mutants. Peaks are labeled as * void; # SERT-GFP; ^ cleaved GFP. (TIF) [file pone.0145688.s001.tif]
